# Identification and characterization of umami peptides from enzymatic hydrolysates of cod skin based on sensory evaluation and molecular simulations

**DOI:** 10.1016/j.fochx.2026.104245

**Published:** 2026-07-23

**Authors:** Shengli Shi, Mengkai Li, Yiqing Wang, Dan Li, Tiantian Wang, Aiguo Luo, Hongmei Zhao, Xiaoli Ma, Yanjun Yang

**Affiliations:** a Department of Biological Science and Technology, Jinzhong University, Jinzhong 030619, China; b Shanxi Composite Condiment Technology Innovation Center, Jinzhong University, Jinzhong 030619, China

**Keywords:** Cod skin enzymatic hydrolysate, Umami peptides, Sensory evaluation, Interactions, Molecular simulation

## Abstract

Cod skin possesses a pronounced savoury flavour yet remains underutilised as a processing by product. As a potential source of umami peptides, cod skin holds significant development potential. This study employed gel permeation chromatography (GPC), ultrafiltration (UF), sensory evaluation, high–performance liquid chromatography tandem mass spectrometry (LC–MS/MS), and virtual screening to isolate, purify, screen, and identify potential umami peptides from cod skin enzymatic hydrolysate (EHCS). Five potential umami peptides: GDRGFPGER, SSGDQGPAG, SSGPFGE, LPGADDDKF, and GLPGADDKF were screened. Their interactions with T1R1/T1R3 receptors and the stability of peptide–receptor complexes were investigated via molecular docking and molecular dynamics simulations. Ultimately, GDRGFPGER was identified as a potent umami peptide, while SSGDQGPAG and SSGPFGE exhibited umami enhancing characteristics. This study provides a screening and identification methodology for extracting umami peptides from cod skin, elucidates the molecular perception mechanism of umami peptides, and establishes a theoretical foundation and technical support for developing natural umami enhancers.

## Highlights

• Five umami peptides were isolated from cod skin enzymatic hydrolysates using GPC, UF, and HPLC-MS/MS.
• Molecular docking and dynamics simulations revealed the interaction and stability of peptide-T1R1/T1R3 receptor complexes.
• GDRGFPGER was identified as a potent umami peptide, with SSGDQGPAG and SSGPFGE also showing umami-enhancing effects.

## 1 Introduction

Umami, one of the five fundamental tastes alongside sweet, sour, bitter and salty, is typically described as a savoury or meaty flavour experience. It primarily derives from amino acids, particularly glutamate, and nucleotides such as inosine monophosphate and guanosine monophosphate, which are widely present in meat, fish, mushrooms and fermented foods (Ramesh et al., 2025). These compounds produce a distinctive taste sensation associated with umami by activating specific receptors on taste buds. Umami peptides are small protein fragments containing glutamate or other flavour–enhancing components, which play a crucial role in regulating the perception of umami (Gu et al., 2024). Following food ingestion, these peptides undergo degradation within the gastrointestinal tract and interact with G protein-coupled receptors (GPCRs), particularly mGluR1 and T1R1/T1R3 receptors, which mediate taste transmission on the tongue (Chen et al., 2025). Activation of these receptors initiates a cascade of intracellular signalling events, thereby enhancing the perception of umami.

Atlantic cod (*Gadus morhua*) ranks among the world's most extensively harvested and economically valuable fish species. However, the utilisation rate of its by product cod skin remains comparatively low. Each year, vast quantities of cod skin are discarded, resulting in substantial resource wastage (Zhao et al., 2024). Cod skin is rich in collagen, amino acids and bioactive peptides, some of which exhibit pronounced umami properties. Consequently, cod skin holds significant development potential as a potential source of umami peptides (Y. Wang et al., 2023b). In recent years, with the growing demand for functional foods, utilising umami peptides from cod skin not only helps reduce resource wastage but also provides the food industry with new natural flavour enhancers (Y. Wang et al., 2023). In particular, umami peptides extracted from cod skin can effectively enhance the savoury flavour of food, making them an ideal source for food additives.

Despite the immense potential of umami peptides in cod skin, existing separation and identification techniques continue to face challenges. Traditional separation methods, such as high performance liquid chromatography, mass spectrometry, and gel filtration chromatography, while capable of extracting some umami peptides from complex aquatic products, typically suffer from operational complexity, low efficiency, and insufficient sensitivity (An et al., 2024; Dutta et al., 2025; X. Wang et al., 2023a). Traditional separation methods struggle to effectively identify low concentration peptide fragments possessing specific umami activity, resulting in the untimely discovery of certain high potential peptides. Consequently, existing separation and identification techniques require further optimisation to enhance the screening efficiency and accuracy of umami peptides. In recent years, researchers have begun applying various computational prediction tools, such as iUmami_SCM, UMPred–FRL, Umami_YYDS, and Umami–MRNN towards predicting umami peptides, establishing relevant methodologies (Lao et al., 2025; Zhang et al., 2024). Concurrently, computational methods such as molecular docking and molecular dynamics simulations have become widely adopted effective approaches for screening umami peptides, alongside studies of umami receptors based on induced fit theory and investigations into the dynamic processes of peptide–receptor interactions (Chen et al., 2024c; Meng et al., 2024). Pan et al. (2025) employed virtual screening techniques combined with molecular docking analysis to identify potential umami peptides derived from soy sauce. By analysing the binding patterns of multiple peptide segments with T1R1/T1R3 receptors, the study predicted their potential umami activity. These peptides umami characteristics were subsequently validated through biosensor and sensory testing. Wei et al. (2025) isolated and characterized novel umami peptides from yellowfin tuna protein hydrolysate, evaluating their taste properties and masking effects on bitter peptides. Utilising molecular docking techniques, he elucidated the molecular mechanism governing the binding of umami peptides to taste receptors. Song et al. (2025) established an efficient, targeted screening system for bonito umami peptides, integrating computational simulation predictions with experimental hydrolysis validation. The research precisely identified potential active peptide segments through computational biology approaches, subsequently verifying their existence and umami activity via experimental hydrolysis and purification. This significantly enhanced the efficiency of umami peptide screening, addressing the long standing challenges of lengthy cycles and broad scope inherent in traditional methods. Hua et al. (2025) established an efficient screening system centred on virtual screening and molecular simulation techniques to identify and characterise natural umami–active components in *Litopenaeus vannamei*. This approach enabled the rapid identification of novel umami peptides previously unreported in this species, thereby filling a research gap in its recognition as a source of umami peptides and enriching the resource pool of umami peptides derived from aquatic sources. However, whilst these computational methods provide crucial theoretical support for umami peptide research, virtual screening–achieved by precisely defining peptide molecular weight distribution ranges and integrating tools such as activity prediction, toxicity assessment, and umami potential forecasting significantly enhances screening efficiency and accuracy when supplemented with molecular docking techniques. Nevertheless, challenges persist in the screening, identification, and screening systems for umami peptides. For instance, computational simulations exhibit inherent uncertainties in predicting peptide–receptor binding affinity, peptide stability, and biological activity. Concurrently, existing research predominantly focuses on short peptide segments ranging from dipeptides to hexapeptides, with comparatively limited systematic attention and in depth exploration of medium length segments such as heptapeptides to nonapeptides. Theoretically, such longer peptides, owing to their richer amino acid composition, may possess stronger umami activity and more complex mechanisms of action. Their umami potential warrants further exploration (Wu et al., 2025).

The T1R1/T1R3 heterodimer is currently recognised as the principal umami taste receptor in mammals and belongs to the G protein-coupled receptor (GPCR) family (Chen et al., 2025; Gong et al., 2026). It consists of two subunits, T1R1 and T1R3, in which the T1R1 subunit is primarily responsible for ligand recognition, while T1R3 functions as an auxiliary subunit involved in signal transduction (Gong et al., 2026; Li et al., 2026; Wang et al., 2025). Extensive studies have demonstrated that glutamate, 5′-nucleotides, and umami peptides from various food sources all bind to the N-terminal domain (NTD) of the T1R1/T1R3 receptor, inducing conformational changes that activate downstream G protein signalling pathways and ultimately generate umami perception (Gong et al., 2026; Wang et al., 2025). Therefore, T1R1/T1R3 serves as one of the most widely used targets in molecular docking and mechanistic studies of umami peptides.

Therefore, the objective of this study was to discover and obtain novel umami peptides from enzymatically hydrolysed cod skin extract (EHCS) from the North Atlantic, and to elucidate the taste mechanism of these umami peptides based on energy interactions and surface forces using molecular docking techniques. We employed a series of sequential experimental and computer–aided approaches, including high performance gel filtration chromatography and ultrafiltration to resolve molecular weight distributions, isolate and purify potential umami peptide fragments, and evaluate the taste of identified peptides through various sensory assessment methods. Furthermore, molecular docking and molecular dynamics simulations (MDS) were employed to investigate interactions between the identified peptides and the T1R1/T1R3 umami receptors. Candidate peptide segments underwent comprehensive evaluation using multiple metrics to screen for potential umami peptides. The application of these technical approaches and methodologies enabled the identification of high umami peptide segments in cod skin and provided support for the application of umami peptides in food additives.

## 2 Materials and methods

### 2.1 Materials and reagents

Formic acid, acetonitrile, flavour protease, trypsin, alkaline protease, neutral protease, composite protease (neutral protease, alkaline protease, and flavourzyme) and other reagents were supplied by Shanghai Maclin Biochemical Technology Co., Ltd. Lysine–lysine–tyrosine–arginine and lysine–lysine–lysine were supplied by Shanghai aladdin biochemical technology co., ltd. All reagents were of analytical grade.

### 2.2 Preparation of EHCS

The preparation method for cod skin enzymatic hydrolysate (EHCS) follows that described in the aforementioned report (Li et al., 2020). Specifically, the cleaned cod skin was cut into 1 cm × 1 cm pieces, followed by vacuum freeze drying for 48 h. The freeze dried cod skin was then ground into powder and stored for subsequent use. 1.0 g of cod skin powder was sieved through an 80 mesh sieve and prepared as a suspension with a substrate concentration of 6.0% (*w*/*v*), with the system pH 6.5. Subsequently, flavour protease was added to the system at a total enzyme loading of 5000 U/g. The reaction mixture underwent enzymatic hydrolysis at 50 °C for 2 h. After cooling, a composite protease was added again, followed by a further 2 h of hydrolysis. Immediately upon completion of the reaction, the mixture was placed in a boiling water bath for 10 min to inactivate the enzymes, then cooled to room temperature. Centrifugation at 8000 r/min and 4 °C for 15 min was performed. The collected supernatant was freeze dried and stored at −80 °C for subsequent use.

### 2.3 Mesuring by GFC

The relative molecular mass and mass distribution of peptides were determined using gel filtration chromatography (GFC) (Agilent 1260 Infinity II), with modifications to the method reported by Gao et al. (2024). Specifically, a TSKgel G2000 SWXL gel chromatography column (300 mm × 7.8 mm, 0.5 μm) was employed. The mobile phase comprised acetonitrile water trifluoroacetic acid (45: 55: 0.1, filtered by a 0.22 μm organic-phase membrane filter and degassed by ultrasonication for 20 min). Detection was performed at 220 nm, with a column temperature of 30 °C, a flow rate of 0.5 mL/min, and an injection volume of 10 μL. 20.0 mg of the aforementioned peptide sample into a 10 mL volumetric flask and sonicate for 10 min. Filter through a 0.22 μm PTFE membrane prior to instrument analysis. Calculate the relative molecular mass of the peptide and its distribution range within the sample.

### 2.4 Ultrafiltration and evaluation

Ultrafiltration was employed for the fractionation of enzymatically hydrolysed samples. Initially, a 0.45 μm microfiltration membrane was used to remove macromolecular impurities, followed by fractionation at molecular weight cutoffs of 1, 3, 5, and 10 kD. Following the reported by Xie et al. (2023), the umami intensity was evaluated using an electronic tongue (Insent TS–5000Z) combined with sensory assessment. The sample exhibiting the highest umami intensity was freeze dried and stored at −80 °C for subsequent use. The electronic tongue testing parameters were as follows: equilibration time 30 s, measurement time 60 s, rinsing time 40 s. Response values were recorded in millivolts (mV), with each sample tested in triplicate.

A trained panel of ten assessors (5 males, 5 females; aged 20–30 years) possessing extensive expertise in sensory evaluation participated in this study. All voluntarily participated after signing informed consent forms. Assessors were instructed to hold each sample in the mouth for 2 min to fully evaluate its flavour profile. Between evaluations, purified water was provided to cleanse the palate. A “sip-and-spit” procedure was used to quantify taste intensity. Reference solutions served as calibration standards (Lao et al., 2025; Xiang et al., 2025). The reference samples for basic tastes were prepared as follows: citric acid (0.08%, *v*/v) for sourness; sucrose (1%, *w*/*v*) for sweetness; L-isoleucine (0.08%, w/v) for bitterness; sodium chloride (0.35%, w/v) for saltiness; monosodium glutamate (MSG, 0.35%, w/v) for umami; and tea polyphenols (0.1%, w/v) for astringency (Lao et al., 2025; Xiang et al., 2025). Samples were rated on a 1-to-10 scale. The reference solution was set at 5. A score of 1 indicated no flavour, and 10 indicated extremely strong taste. Scoring criteria for both electronic tongue and sensory evaluation were quantified according to Tables S1 and S2 respectively.

### 2.5 Identification of peptides in EHCS

Following the reported by Shen et al. (2022), the obtained peptide samples were first subjected to desalting treatment. Specifically, 3 mL sample to be analysed was desalted using an Empore solid phase extraction column C18–SD. The desalted sample was freeze dried and subsequently resuspended in 40 μL of 0.1% formic acid. Peptide separation was performed using a Zorbax 300SB–C18 reverse phase column (75 μm × 150 mm, 3 μm), maintained at room temperature with a flow rate of 250 nL/min. The mobile phase consisted of phase A (0.1% formic acid aqueous solution) and phase B (0.1% formic acid–acetonitrile aqueous solution, 8% acetonitrile by volume). The elution gradient was set as: 0–50 min, phase A decreased from 96% to 50%, phase B increased from 4% to 50%. From 50 to 54 min, phase A decreased from 50% to 0%, while phase B increased from 50% to 100%. From 54 to 60 min, the elution conditions of 0% phase A + 100% phase B were maintained to thoroughly flush residual components from the column.

The separated peptide samples were subjected to mass spectrometry analysis in positive ion mode. The primary mass spectrometry parameters were set as follows: *m*/*z* scan range 300–1800, resolution 70,000, automatic gain control (AGC) target value 1e6, maximum ion injection time 10 ms, scan range count 1, dynamic exclusion time 20.0 s. Secondary mass spectrometry employed a data dependent acquisition (DDA) strategy with a resolution of 17,500, micro scan count of 1, maximum ion injection time of 60 ms, normalised collision energy of 27 eV, and minimum fill ratio of 0.1% to ensure the integrity and accuracy of fragment ion signals. The limit of detection (LOD) and limit of quantification (LOQ) were determined based on signal-to-noise ratios of 3 and 10, respectively, and were calculated to be 0.02 μg/mL and 0.06 μg/mL.

Raw mass spectrometry data were analysed using MaxQuant 1.5.5.1 software against the UniProt–Actinopterygii database. Search parameters were set as follows: primary mass spectrometry mass tolerance ≤20 ppm, secondary mass spectrometry mass tolerance ≤0.02 Da. Enzymatic digestion was configured as non restrictive, permitting a maximum of two missed cleavage sites. Dynamic modification was set to methionine (M) oxidation. To control false positives in identification, the confidence threshold for both peptide and protein levels was set to a false discovery rate (FDR) ≤ 0.01. Final qualitative identification of peptides was achieved based on matched peptide information.

### 2.6 Virtual screening

Following the reported by Gu et al. (2025), the virtual screening of umami peptides was conducted using integrated machine learning technologies (UMPred–FRL, Umami–YYDS, Taste Peptides DM). Peptide segments obtained through the aforementioned screening process must meet the following criteria: bioactivity score > 0.5, non toxicity, good water solubility, and predicted score > 0.6. These selected peptide segments, as potential umami peptides, undergo further validation through static molecular docking and dynamic molecular dynamics simulations.

### 2.7 Homology modeling and molecular docking

The data for T1R1 (Q7RTX1) and T1R3 (Q7RYX0) were obtained from the UniProtKB database. To construct homology models of the umami receptors T1R1 and T1R3, the metabolic glutamate receptor (PDB ID 1EWK) was utilised as a structural template for model construction, optimisation and evaluation on the SWISS-MODEL online platform (https://swissmodel.expasy.org/) (Xiang et al., 2025). To evaluate the model, Ramachandran plots were generated using SAVESv 6.0 and subsequently analysed to assess the distribution of amino acids across the optimal, permitted, tolerance and non permitted regions. It was determined that the T1R1/T1R3 receptor model must be at least 90% in the most favourable regions to ensure its structural validity (Shen et al., 2025). Using the DoG Site Scorer module within the POCASA online platform, binding pocket predictions were conducted for the T1R1/T1R3 umami receptors. Screening criteria were set as a drug binding affinity score ≥ 0.7 and a simplified score ≥ 0.5, with results utilised for subsequent molecular docking studies (Huang et al., 2024).

The three-dimensional structures of the potential umami peptides used for molecular docking were generated based on the amino acid sequences identified by LC-MS/MS. Briefly, the two-dimensional structures of the five target peptides were drawn using ChemDraw 2024 and subsequently imported into Chem3D 2024 for structural optimization. Energy minimization was performed using the MMFF94 force field with the steepest descent and conjugate gradient algorithms. The convergence criterion was set to an energy gradient of <0.001 kcal/(mol·Å), and the maximum number of optimization steps was set to 10,000. The optimized low-energy conformations were saved in PDB format and used as ligand structures for subsequent molecular docking analysis (Feng et al., 2024; Gao et al., 2024).

AutoDock Vina software was employed to generate docking models of the T1R1/T1R3-EHCS complex, with the umami enhancing peptide docked to form a novel ternary system model. Comprehensive analysis was conducted on the binding energy and interaction patterns of T1R1/T1R3. Three dimensional structural visualisation analysis was performed using PyMOL software, with a focus on elucidating key hydrogen bond interactions within the ligand receptor complex. Discovery studio software generated two dimensional interaction maps to systematically identify and quantify non covalent interaction types, including hydrogen bonds, hydrophobic interactions, π–π stacking, and electrostatic forces. This enabled a comprehensive analysis of the molecular recognition mechanism between the potential umami peptide and the T1R1/T1R3 receptors (Feng et al., 2024).

### 2.8 Molecular dynamics simulations (MDS)

Using the Sobtop–1.0 (dev 3.1) platform, generate ligand topology files based on the AMBER force field. Receptor ligand complexes generated via molecular docking were analysed using MDS within GROMACS 2024, employing the amber 99sb.ff force field for proteins and the GAFF force field for ligands. Prior to molecular dynamics simulations, comprehensive preparation was conducted for all systems including ions, solvents, receptors, and ligands through energy minimisation using the steepest descent algorithm (Lao et al., 2025). Using an NVT (number of atoms, volume, temperature) ensemble, the structure was stabilised via 100 ps of time stepping simulations. Subsequently, an NPT (number of atoms, pressure, temperature) ensemble was applied, equilibrating for 100 ps under constant pressure (1 Pa) and temperature (300*K*). The production phase of the molecular dynamics simulations spanned 100 ns. Additionally, GROMACS was employed to compute kinetic analyses including root mean square deviation (RMSD), root mean square fluctuation (RMSF), radius of gyration (Rg), Gibbs free energy landscape, and hydrogen bond counts from the trajectories (Xiang et al., 2025).

### 2.9 Sensory analysis of synthetic peptides

Following the reported by Chen et al. (2023), the umami peptide solutions were prepared and synthesized, subsequently undergoing serial dilutions with ultrapure water at a 1:1 ratio, with deionised water serving as the control. Sensory evaluation and electronic tongue taste assessment of the 5 umami peptides were conducted in accordance with section 2.4.

### 2.10 Statistical analysis

Data were obtained by averaging results from three parallel experiments and are presented as mean ± standard deviation (SD). Significant differences between samples were determined using analysis of variance (ANOVA), whilst statistical significance testing was conducted at a 95% confidence interval using SPSS software (version 22.0).

## 3 Results and discussion

### 3.1 Analysis of peptide isolation and sensory evaluations in EHCS

**Fig. 1:**
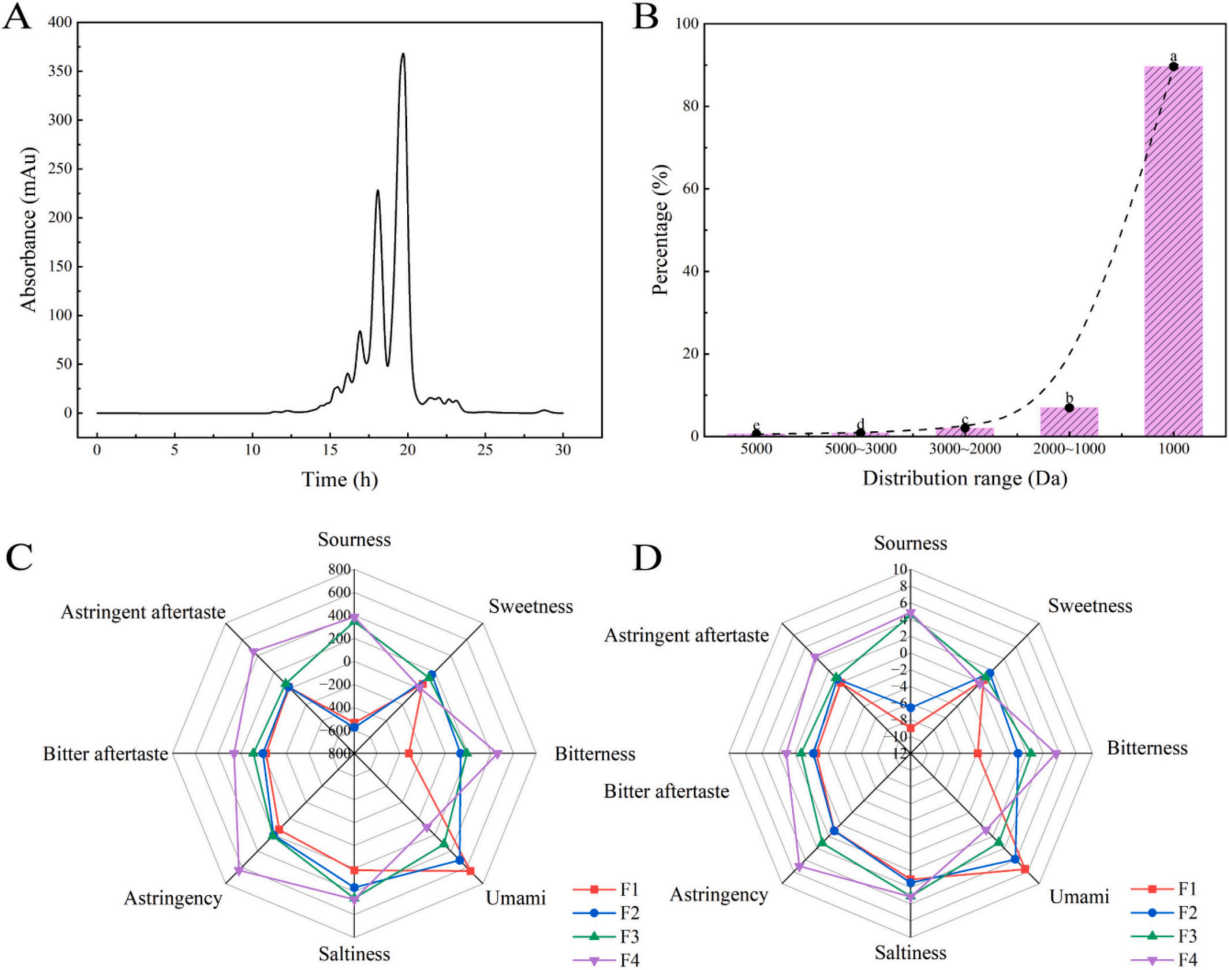
Gel permeation chromatography analysis and sensory evaluation. GPC of the UF fraction (A); Molecular weight distribution of the peptides (B); Electronic tongue analysis of the peptides, different letters mean significant differences (*p* < 0.05) (C); Sensory analysis of the peptides (D).

Starting from 1.0 g of dried cod skin powder, a total peptide powder of 0.187 ± 0.012 g was obtained after sequential enzymatic hydrolysis, centrifugation, ultrafiltration, and freeze-drying, corresponding to an overall peptide yield of 18.7% (*w*/w). Through GPC analysis, the molecular weight distribution of peptides in EHCS was determined (Fig. 1A), with five characteristic peaks isolated. Further analysis of the molecular weight ranges corresponding to each peak (Fig. 1B) revealed that peptides with molecular weights below 1000 Da constituted 89.63% of the total (*P* < 0.05), significantly higher than peptides in other molecular weight ranges. Consequently, peptides within this molecular weight range are likely key components of the umami peptides in cod skin.

Ultrafiltration was employed to fractionate EHCS samples, yielding five distinct fractions: F1: <1000 Da, F2: 1000–2000 Da, F3: 2000–3000 Da, F4: 3000–5000 Da, and F5: >5000 Da. The taste profiles of F1–F4 were characterized using electronic tongue analysis and human sensory evaluation. As shown in Fig. 1C, F1 exhibited the most pronounced umami profile, with its electronic tongue umami intensity response value significantly higher than other fractions (*P* < 0.05, Table S3). Its umami intensity was approximately 1.3 times that of F2, 1.8 times that of F3, and 2.2 times that of F4. Combined with molecular weight distribution analysis, the results suggest that peptide segments below 1000 Da, being more readily and efficiently bound to the umami receptors T1R1/T1R3, serve as the core carriers for umami-active fragments, including umami peptides. Concurrently, the response values for sourness, astringency, and bitter aftertaste dimensions in F1 were lower than in other fractions, suggesting a relatively clean taste profile with limited undesirable taste attributes. This may be associated with the reduced contribution of larger peptide fractions, which are often related to bitterness generation during incomplete hydrolysis (Wei et al., 2025). Furthermore, consistent with literature findings indicating umami peptides predominantly cluster below 1000 Da, this may stem from superior solubility and dispersibility of such small molecule peptides within the system. Compared to other peptide size ranges, they facilitate easier contact with taste buds, more readily activate umami receptors, and enhance umami perception (Hossain et al., 2024). Fig. 1D presents the results of the human sensory evaluation for F1–F4. Among the tested fractions, F1 exhibited a significantly higher umami intensity score than the others (P < 0.05, Table S3), while displaying minimal negative taste attributes. These findings identify F1 as the most promising umami peptide active fraction in EHCS, owing to its superior umami intensity and limited undesirable flavour, making it an ideal candidate for umami peptide identification and subsequent experimental studies.

### 3.2 Identification and prediction screening of umami peptides in EHCS

A total of 1316 peptide fragments were identified from the aforementioned F1 fraction. To screen for umami peptides with application potential, a multi dimensional screening strategy was employed. Relevant reports indicate that umami peptides typically comprise 10 or fewer amino acids. The PeptideRanker score indicates biological activity potential, with scores above 0.5 indicating high biological activity (Chen et al., 2024a). Based on this, the PeptideRanker tool was employed to screen for 527 peptide segments with a biological activity score > 0.5. Toxicity assessment was conducted using the Toxin Pred tool, retaining 316 safe candidate peptides after excluding potentially toxic segments. Further characterization of water solubility was performed using the Innovagen tool, yielding 31 peptides with favourable water solubility.

A further selection of 7–9 peptides (11 in total) was subjected to evaluation using three independent umami peptide prediction tools (UMPred–FRL, Umami–YYDS, TastePeptidesDM). The evaluation scores were normalised using the Min–Max method, and the peptides were ranked according to their composite scores (Table 1). This resulted in the identification of five potential umami peptides (LPGADDKF, GDRGFPGER, SSGPFGE, GLPGADDKF, SSGDQGPAG) with composite scores greater than 0.9.

**Table 1:** Prediction screening results of potential umami peptides.

Rank	peptides	toxicity	Water solubility	UMPred–FRL score	Umami–YYDS Score	TastePeptidesDM Score	Composite scores
1	LPGADDKF	None	Good	0.786	1.000	0.977	0.997
2	GDRGFPGER	None	Good	0.732	1.000	0.983	0.976
3	SSGPFGE	None	Good	0.743	0.990	0.980	0.972
4	GLPGADDKF	None	Good	0.665	1.000	0.980	0.945
5	SSGDQGPAG	None	Good	0.578	1.000	0.975	0.904
6	AGPAGPR	None	Good	0.092	1.000	0.974	0.688
7	APGPVGPA	None	Good	0.035	1.000	0.940	0.649
8	GPSPTWPQ	None	Good	0.059	0.999	0.816	0.607
9	GSSGPFG	None	Good	0.049	1.000	0.188	0.340
10	GPAGPSGK	None	Good	0.080	0.932	0.250	0.330
11	ATGPSGF	None	Good	0.058	0.536	0.878	0.300

**Fig. 2:**
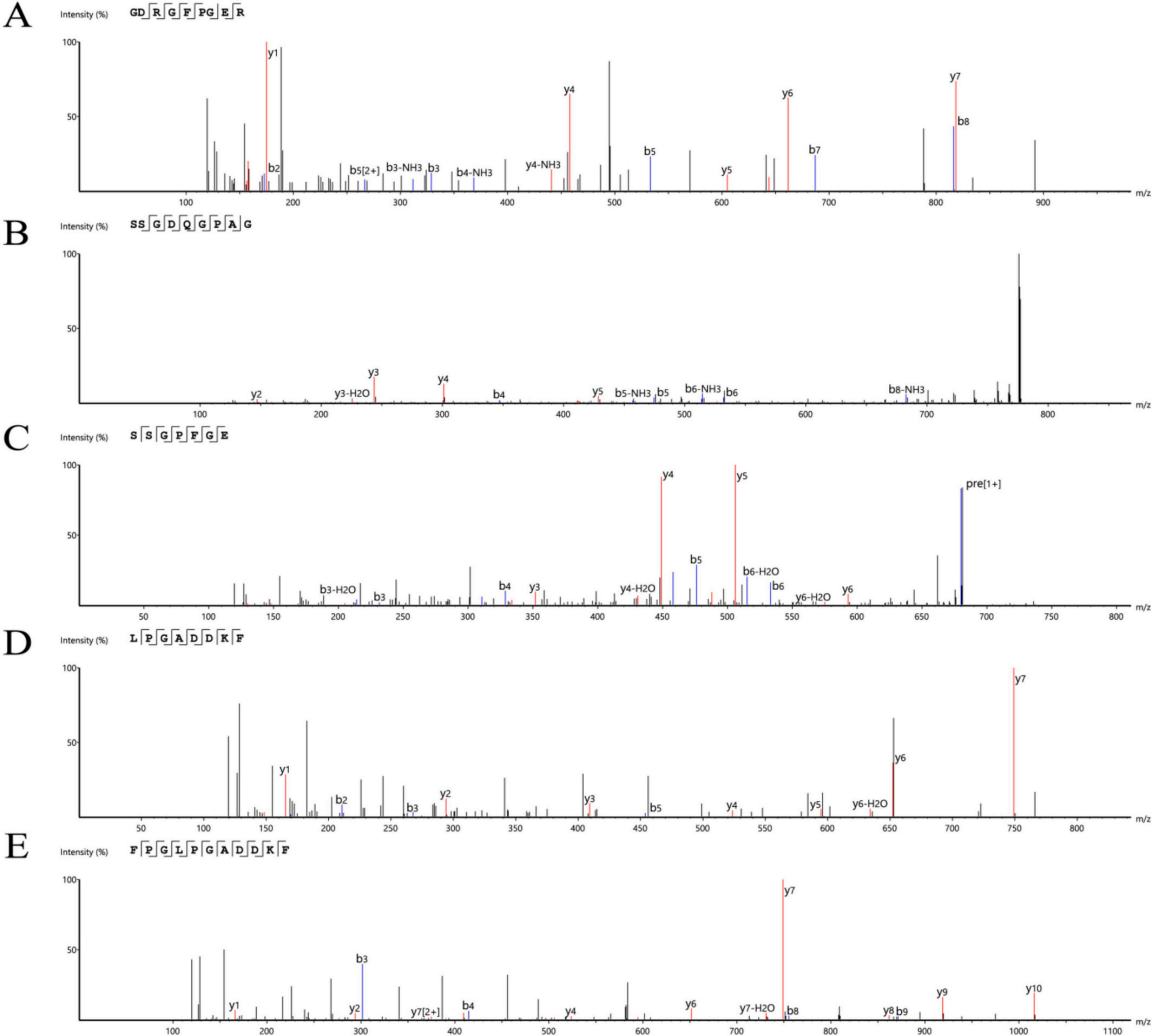
Secondary mass spectra of potential umami peptides.

The five potential umami peptides passed screening, likely owing to their functional amino acid residues such as glutamic acid and aspartic acid, which readily adopt active conformations at umami taste receptors, while lacking toxic residues like citrulline and homocysteine. Additionally, these peptides are rich in polar amino acids such as glycine, aspartic acid, and glutamine, whose hydrophilic side chains enhance solubility (Cui et al., 2024). The five potential umami peptides identified through screening serve as priority candidate peptide segments for subsequent experimental validation, with their mass spectra shown in Fig. 2.

### 3.3 Molecular docking of umami peptides to the umami receptor T1R1/ T1R3

**Fig. 3:**
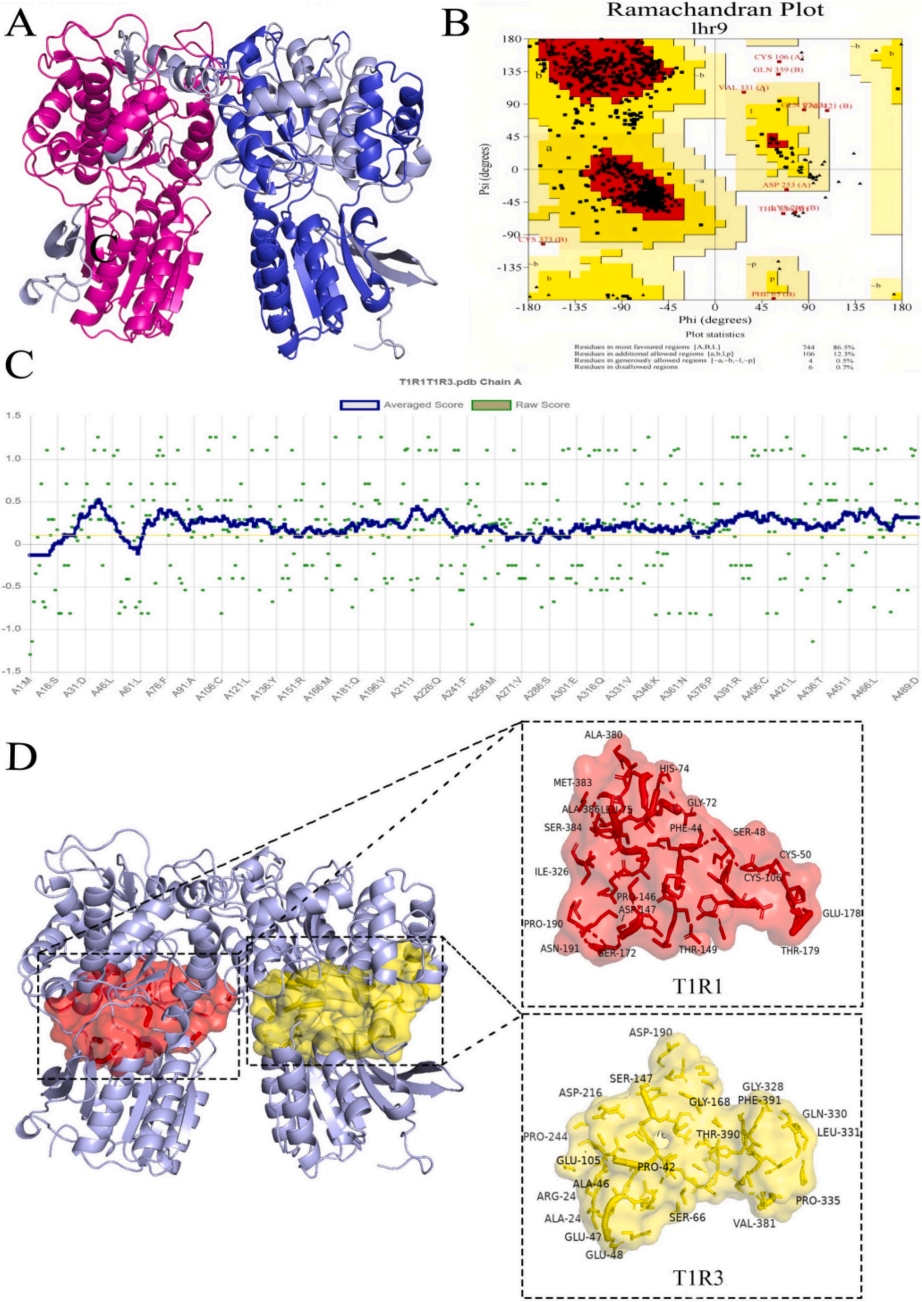
Predicted umami receptors structure of T1R1/T1R3. Homology model of receptor T1R1/T1R3 (A); Rasch diagram of homology modeling of T1R1/T1R3 receptor (B); Structural assessment score chart of T1R1/T1R3 receptor (C); Predicted active binding pockets of T1R1/T1R3 receptor (D).

Modeling results from the SWISS-MODEL online platform reveal an umami receptor model with an amino acid sequence coverage exceeding 0.85 (Fig. 3A). Fig. 3A shows that T1R1/T1R3 as a whole exhibits a dumbbell like structure, with T1R1 adopting a closed conformation and T1R3 an open conformation; its ligand-binding domain resembles the shape of a Venus flytrap. The validity of the T1R1/T1R3 umami receptor model was comprehensively assessed using Rasmuson and Verify 3D evaluations. As shown in Fig. 3B, 99.3% of the protein model's Rasmuson scores fell within the permissible range, with only 0.7% outside this range, meeting the standard criterion for Rasmuson protein model evaluation (≥95%). Fig. 3C indicates that 86.2% of amino acid residues in the Verify 3D protein evaluation scored above 0.2, meeting the general standard for Verify 3D model evaluation (>80%). The combined results from the Ramachandran plot and Verify 3D evaluation demonstrate that the constructed homology model is reasonable and effective, providing a suitable receptor protein for subsequent molecular docking.

Fig. 3D depicts two predicted active pockets for the T1R1/T1R3 umami receptor, located within the T1R1 subunit (red region) and T1R3 subunit (yellow region) respectively. Their drug binding capacities are 0.81 and 0.8, with simplified scores of 0.6 and 0.61. As shown in Table S4, the binding pocket within the T1R1 subunit has a volume of 1748.91 Å^3^, surface area of 1894.35 Å^2^, depth of 27.6 Å, spatial coordinates ranging from x:40, y:30, z:24, with central coordinates −18.348, 6.164, 39.699. This pocket is formed by residues Ala 380, Met 383, Ala 386, Leu 78, Ser 384, Ile 326, Pro 146, Asp 147, Pro 190, Asn 191, His 74, Gly 72, Phe 44, Ser 46, Cys 50, Cys 106, Glu 178, Thr 149, Thr 199, Glu 172, Asp 218, Ser 248, Phe 247, Arg 151. The binding pocket within the T1R3 subunit has a volume of 1386.83 Å^3^, a surface area of 1373.27 Å^2^, and a depth of 22.8 Å. Its coordinate range is x: 42, y: 36, z: 24, with central coordinates at 13.777, −14.003, 38.51. The T1R1 pocket exhibits a 26.1% larger volume and 21.1% greater depth than T1R3, with higher hydrogen bond acceptors and donors compared to the T1R3 binding pocket. This phenomenon likely arises because T1R1 serves as the primary recognition subunit, while T1R3 functions as an auxiliary recognition subunit binding pocket, consistent with other published reports (Gu et al., 2025; Pan et al., 2025).

As demonstrated by Belloir et al. (2017), through in vitro expression and purification of feline T1R1–NTD, it was established that this domain constitutes the primary recognition site for umami ligands, capable of independently binding diverse amino acids and nucleotides whilst exhibiting synergistic effects with IMP. These findings provide direct biochemical evidence for T1R1 serving as the primary recognising subunit of the umami receptor, requiring an internal structure capable of accommodating and recognising diverse umami binding moieties. T1R1 comprises Asp 190, Asp 216, Ser 147, Pro 244, Glu 105, Ala 46, Arg 24, Ala 24, Glu 47, Glu 48, Pro 42, Gly 328, Phe 391, Gln 330, Leu 331, Thr 390, Pro 335, Ser 66, Val 381, Ser 306, Leu 468, Asn 386, and Gln 330. These geometric features reflect the binding pocket's spatial capacity, interface breadth, and stability. The larger volume of the T1R1 pocket may accommodate peptide segments adopting a wider range of conformations, while its greater depth potentially enhances interaction strength. This provides a basis for subsequent single subunit molecular docking to validate the primary and auxiliary recognition subunits (Yang et al., 2022; Zhang et al., 2019). Concurrently, Liu et al. (2019) elucidated the molecular mechanism by which T1R1–T1R3 recognise umami ligands at the atomic level through molecular dynamics simulations. This revealed the functional characteristics of T1R1 as the primary recognition subunit and the fundamental nature of the ligand structure activity relationship.

**Fig. 4:**
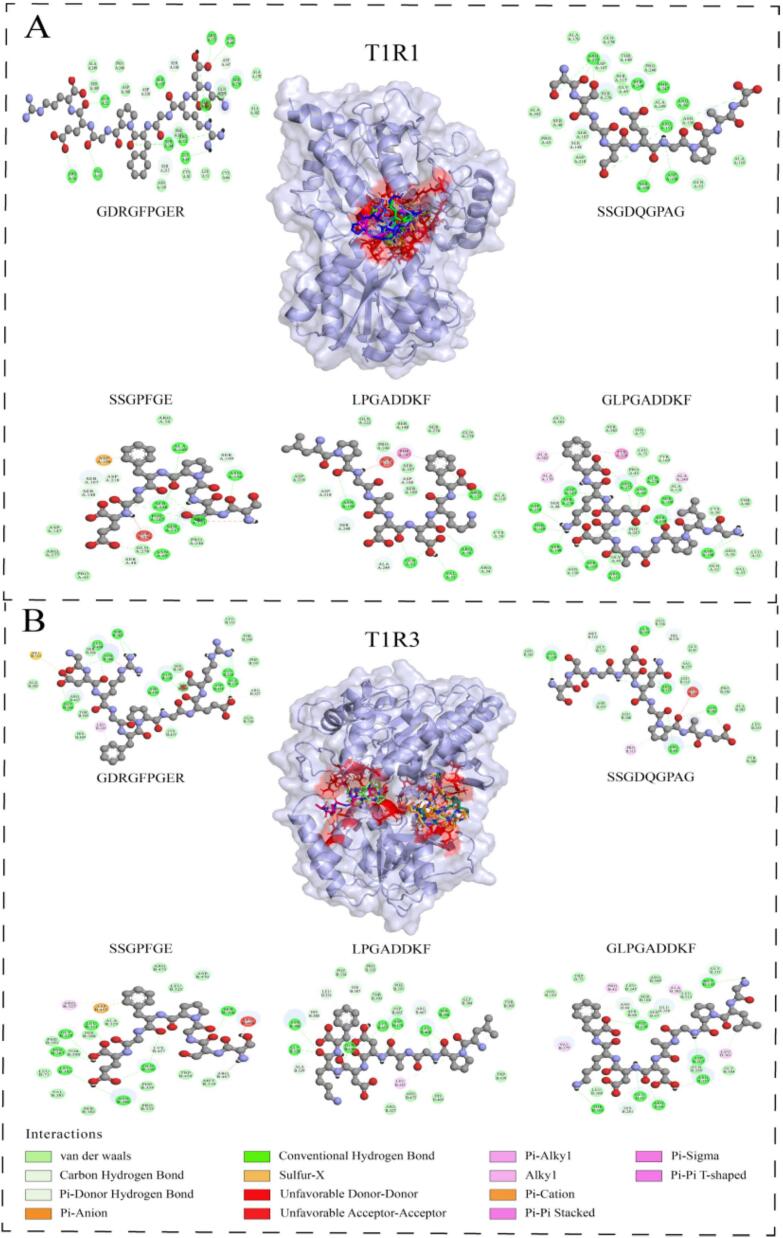
Molecular docking analysis of umami peptides GDRGFPGER, SSGDQGPAG, SSGPFGE, LPGADDKF, and GLPGADDKF with umami receptors T1R1 (A) and T1R3 (B).

Fig. 4A and B respectively depict the optimal docking conformations of five potential umami peptides: GDRGFPGER, SSGDQGPAG, SSGPFGE, LPGADDKF, and GLPGADDKF with the T1R1/T1R3 monomer subunits of the umami receptor, as determined via molecular docking techniques. These illustrations reveal the ideal binding configurations between receptor and ligand. As shown in Table 2, the molecular docking energies between umami peptides and the T1R1 receptor ranged from −7.006 to −8.404 kcal/mol, while those with the T1R3 receptor ranged from −6.771 to −7.161 kcal/mol. Lower docking energies typically indicate stronger bonding between ligand and receptor, resulting in more stable complexes (Zhuang et al., 2025). Therefore, five peptide segments can form stable complexes with both the T1R1 and T1R3 subunits. Free energy analysis further indicates that the binding free energies of peptides with T1R1 subunit are superior to those with T1R3 subunit, with differences ranging from 0.085 to 1.633 kcal/mol. This indicates that umami peptides exhibit distinct subunit selectivity when docking with umami receptors. This finding aligns with the classical theory positing that T1R1 serves as the primary recognition subunit, while T1R3 functions as an auxiliary recognition subunit (Wang et al., 2025). Table S5 indicates the key amino acid residues involved in the molecular docking between potential umami peptides and T1R1/T1R3 receptors. Asp 218, Ser 248, Phe 247 and Arg 151 participate in all interactions between the T1R1 subunit and potential umami peptides. In the interaction between T1R3 and potential umami peptides, Ser 306, Leu 468, Asn 386 and Gln 330 make significant contributions.

**Table 2:** Molecular docking energy between umami peptides and T1R1/T1R3 receptors.

Peptides	T1R1(kcal/mol)	T1R3 (kcal/mol)	T1R1/T1R3 (kcal/mol)	Difference (kcal/mol)
GDRGFPGER	−8.404	−6.771	−9.85	1.633
SSGDQGPAG	−8.017	−6.776	−9.081	1.247
SSGPFGE	−7.251	−7.079	−7.846	0.172
LPGADDKF	−7.006	−6.769	−8.326	0.329
GLPGADDKF	−7.246	−7.161	−8.455	0.085

**Fig. 5:**
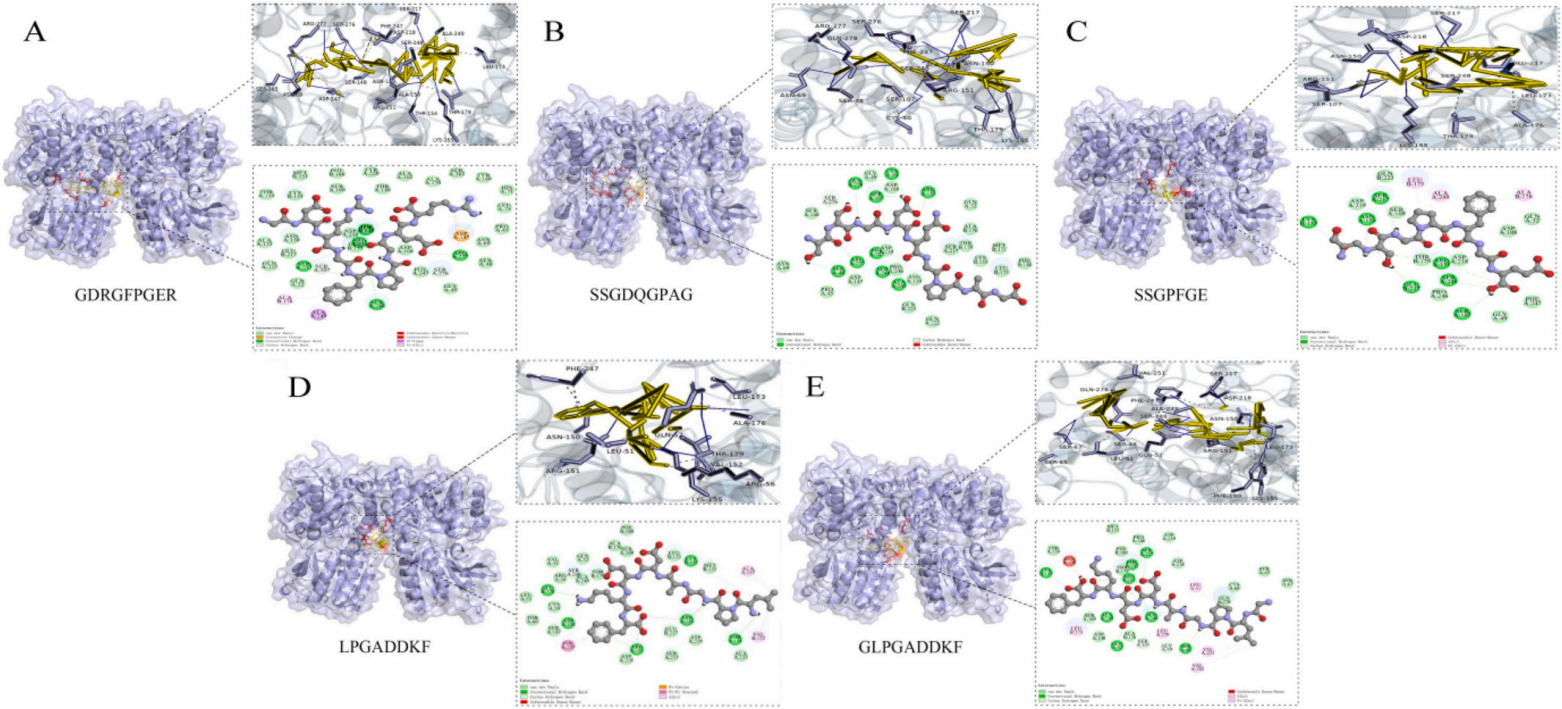
Molecular docking analysis of umami peptides GDRGFPGER (A); SSGDQGPAG (B); SSGPFGE (C); LPGADDKF (D); and GLPGADDKF (E) with umami receptors T1R1/T1R3.

Building upon the docking of the monomeric umami receptors T1R1/T1R3, molecular docking was performed for five potential umami peptides with the T1R1/T1R3 heterodimer. As shown in Fig. 5, five potential umami peptides formed stable complexes with the T1R1/T1R3 heterodimer. The binding free energies of T1R1/T1R3 heterodimer were superior to those of the monomers. This phenomenon may arise from the synergistic effect of T1R3, as an auxiliary recognition subunit, with T1R1, the primary recognition subunit, during the binding process. This aligns with the findings of Hu et al. (2025), who provided direct evidence for the superior binding free energy of T1R1/T1R3 heterodimers over monomers through molecular dynamics simulations and free energy calculations.

Although T1R3 exhibits extremely low affinity for glutamate, it is essential for high affinity dimeric binding. By inducing conformational optimisation of T1R1 and providing structural support, it enables the functional advantages of the heterodimer. Three dimensional (3D) docking simulations revealed the ideal binding conformation between receptor and ligand, highlighting key active sites. This visualisation provides a crucial foundation for investigating the interplay between these two components. Analysis indicates that five umami peptides bind to the pocket domain of the T1R1/T1R3 heterodimer, suggesting their potential for receptor recognition and formation of stable receptor protein complexes. The docking energy parameters for five umami peptides with the T1R1/T1R3 heterodimer in Table 2 indicate that the absolute binding energies of potential umami peptides with heterodimer are significantly higher than those with the T1R1 monomer. This suggests greater binding stability of the peptides with heterodimer. Table S6 details the interaction relationships: five potential umami peptides interact with key recognition amino acid residues of the T1R1 monomer (Asp 218, Ser 248, Phe 247, Arg 151, etc.), while simultaneously interacting with residues on the T1R1 A chain (Ala 249, Asn 150, Ser 217) and the T1R1 B chain (Leu 173, Thr 179, and Lys 155 of the T1R1 B chain. These residues all significantly contribute to peptide receptor binding. The results indicate that during the binding of umami peptides to the T1R1/T1R3 umami receptors, the T1R1 subunit serves as the primary recognition unit, achieving core recognition through interactions with multiple amino acid residues on this subunit. The T1R3 subunit plays an auxiliary recognition role, further enhancing the binding stability of the peptide receptor complex by providing some of the amino acid residues involved in the interactions (Mei et al., 2024).

### 3.4 MDS analysis

Molecular docking can yield conformations of intermolecular interactions, however, it solely considers interactions between flexible ligands and rigid receptors, presenting certain limitations in evaluating the binding stability between ligands and receptors (Jia et al., 2023). Based on, to evaluate the dynamic binding characteristics and stability of five potential umami peptides: GDRGFPGER, GLPGADDKF, LPGADDKF, SSGDQGPAG, and SSGPFGE forming complexes with T1R1/T1R3 umami receptors, molecular dynamics simulations lasting 100 ns were conducted for each system. By analysing multidimensional parameters including root mean square deviation, radius of gyration, root mean square fluctuation, Gibbs free energy landscape, and hydrogen bond count, a comprehensive evaluation was conducted on the structural stability, local flexibility, thermodynamic preference, and molecular interaction basis of each complex.

**Fig. 6:**
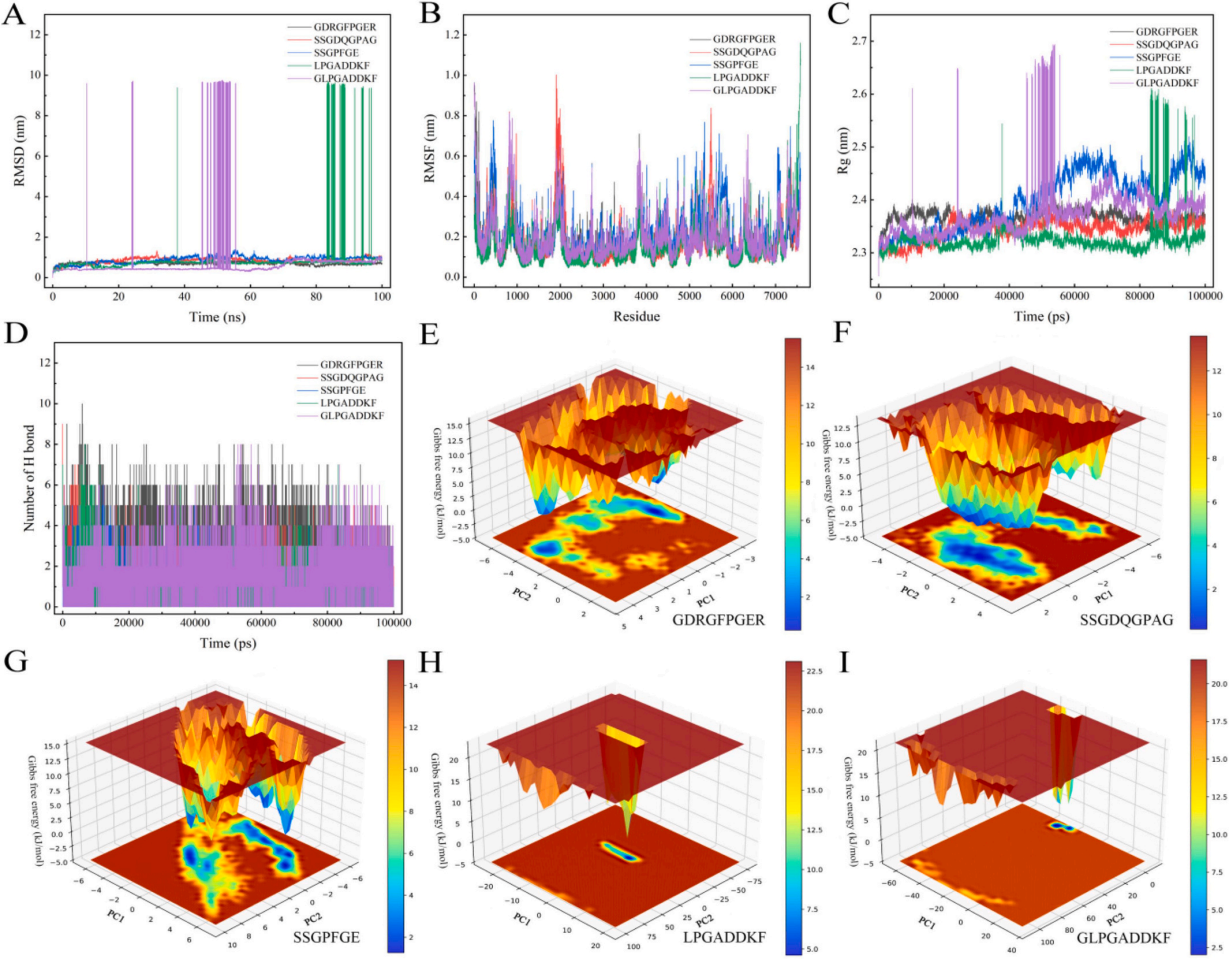
Molecular dynamics simulations and free energy of T1R1/T1R3 analysis. Changes in root mean aquare deviation (RMSD) (A); Root mean square fluctuation (RMSF) (B); Radius of Gyration (Rg) (C); H–bond number (D); Changes in free energy of T1R1/T1R3 with GDRGFPGER (E); SSGDQGPAG (F); SSGPFGE (G); LPGADDKF (H); GLPGADDKF (I).

MDS simulation results indicate that the stability and interaction characteristics of five umami peptides with the T1R1/T1R3 heterodimer complex exhibit significant differences. As shown in Fig. 6A, the RMSD of the T1R1/T1R3 umami receptor protein scaffold stabilised between 0.1 and 0.4 nm during the later stages of simulation, with no irreversible conformational drift or unfolding, demonstrating excellent structural stability. Fig. 6B and C indicate that complex stability can be categorised into two classes: GDRGFPGER–T1R1/T1R3 complex exhibited RMSD fluctuations between 0.6 and 0.8 nm, with a radius of gyration (Rg) maintained at 2.4–2.5 nm. SSGDQGPAG–T1R1/T1R3 complex showed RMSD fluctuations between 0.6 and 1.2 nm without a sustained upward trend. Both complexes demonstrated robust structural dynamic equilibrium and overall stability. The atomic RMSF of GDRGFPGER–receptor system fluctuated between 0.2 and 1.0 nm. In the SSGDQGPAG–receptor system, only atom 2000 exhibited a single peak, with the remainder fluctuating between 0.2 and 0.8 nm, indicating local flexibility within the normal range. Fig. 6D illustrates the temporal variation in hydrogen bond counts. Results indicate that GDRGFPGER, SSGDQGPAG–T1R1/T1R3 complexes, and their respective hydrogen bond counts range from 25 to 30. Close proximity atomic pairs exhibit minimal hydrogen bond distance differences, reflecting high hydrogen bond formation efficiency, strong structural complementarity, and stable conformational dynamics. Conversely, SSGPFGE, LPGADDKF, and GLPGADDKF complexes with the receptor exhibited poor stability. The former two displayed pronounced RMSD fluctuations, while LPGADDKF and GLPGADDKF complexes exhibited a sharp RMSD spike reaching 10 nm. Rg analysis revealed that the total Rg of SSGPFGE complex steadily increased from 2.3 nm to over 2.5 nm. while the latter two exhibited abrupt R_g surges at 80,000 ps and 20,000, 40,000, and 60,000 ps, respectively. This indicates structural loosening and potential conformational collapse or peptide dissociation. Regarding local flexibility, the RMSF of the LPGADDKF–receptor system surged abruptly beyond 1.2 nm after atom 7000 and exhibited an upward trend, indicating pathological hyperflexibility. GLPGADDKF receptor system exhibits multiple local RMSF peaks, all below 1.0 nm. Hydrogen bond analysis reveals both systems possess approximately 80 hydrogen bonds, yet their formation ratio is low. The proximity atom pair curve exhibits significant fluctuations, indicating poor interaction stability. This may be accompanied by conformational changes or dissociation following transient contact at the binding site. Therefore, among five potential umami peptides: GDRGFPGER, SSGDQGPAG, SSGPFGE, LPGADDKF, and GLPGADDKF–SSGPFGE, LPGADDKF, and exhibit poor conformational stability and low hydrogen bond formation efficiency during dynamic binding. Conversely, GDRGFPGER and SSGDQGPAG maintain overall conformational stability with high hydrogen bond efficiency, showing no sustained spikes or disintegration, with GDRGFPGER demonstrating significantly higher stability than the other umami peptides. This phenomenon may stem from the limited and dispersed number of rigid residues in SSGPFGE, LPGADDKF, and GLPGADDKF, coupled with contiguous flexible residues. This configuration permits unrestricted free rotation of the peptide chain, leading to heightened conformational instability. Conversely, GDRGFPGER and SSGDQGPAG feature rationally distributed rigid residues (particularly proline), effectively constraining excessive chain flexibility. Their moderate flexible residues maintain dynamic conformational equilibrium, while balanced hydrophobic and hydrophilic distribution enables hydrophobic residues to form a compact core, underpinning structural stability (Hua et al., 2025).

In molecular dynamics simulations, free energy landscape plots have proven to be a powerful tool for revealing the structural stability of coordination complexes, where strong interactions lead to the formation of distinct, smooth minimum energy regions within the energy distribution (Chen et al., 2024b). Fig. 6 (E–I) depicts the free energy landscape maps of five potential umami peptides interacting with their receptors, as simulated by molecular dynamics. The most stable structures are indicated by purple and blue spots, signifying the lowest energy values. As depicted in Fig. 6 (E–G), molecular dynamics simulations reveal that the GDRGFPGER–T1R1/T1R3 complex exhibits multiple blue low free energy regions within the free energy landscape analysis. These correspond to several stable conformations, represented by distinct “energy valleys”. The free energy differences (valley heights) are moderately sized, with clearly defined stable regions, indicating overall good conformational stability. In contrast, the SSGDQGPAG–T1R1/T1R3 complex exhibits two clusters of blue low energy regions in the free energy landscape analysis. The depth of these “energy valleys” is effectively moderate, significantly constraining conformational changes and indicating overall good conformational stability. Similarly, the SSGPFGE–T1R1/T1R3 complex exhibits two clusters of blue low free energy regions in its free energy landscape analysis, which substantially constrain conformational changes. The moderate depth of the “energy valleys” maintains the overall conformation within a stable range. Fig. 6 (H—I) molecular dynamics simulation results indicate that both the LPGADDKF–T1R1/T1R3 and GLPGADDKF–T1R1/T1R3 complexes exhibit severely inadequate molecular conformational fit between the umami peptide and the T1R1/T1R3 umami receptor, resulting in poor overall conformational stability.

Molecular dynamics simulations further confirmed the favourable stability of complexes formed between T1R1/T1R3 receptors and two potential umami peptides. This demonstrates that the umami peptides GDRGFPGER and SSGDQGPAG can bind to the umami receptors T1R1/T1R3, and that the complexes they form exhibit good conformational stability.

### 3.5 Evaluation of synthesized peptides

**Fig. 7:**
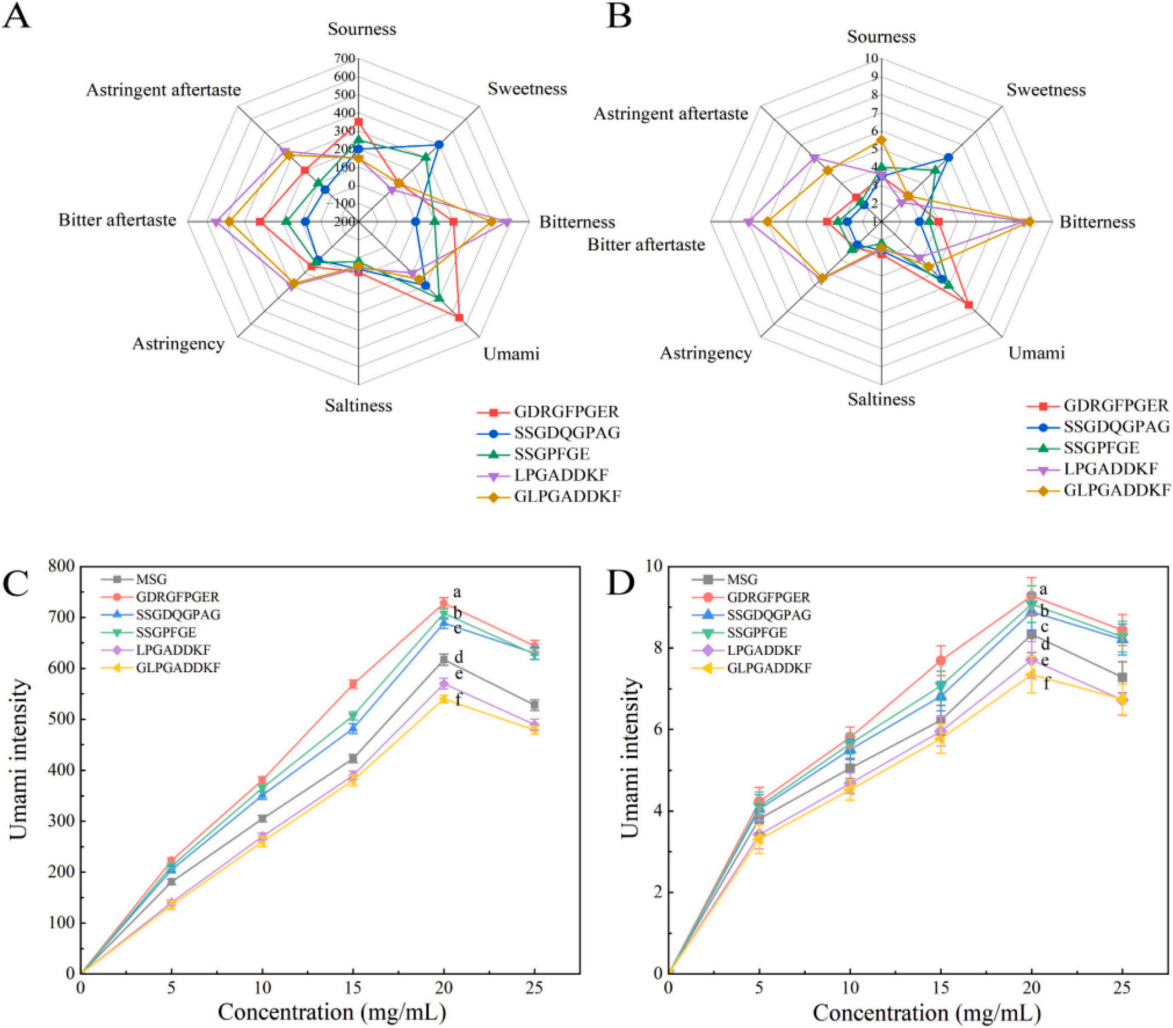
Sensory evaluation of synthetic peptides (A); Electronic tongue analysis of synthetic peptides (B); Sensory evaluation of synthetic peptides with MSG (C); Electronic tongue analysis of synthetic peptides with MSG (D). Different letters mean significant differences (*p* < 0.05).

To evaluate the umami intensity of five potential umami peptides in practice, synthetic analyses were conducted on these peptides, as shown in Fig. 7A and B. Both electronic tongue and human sensory evaluation results revealed significant taste profile heterogeneity among the five umami peptides, categorisable into two groups: GDRGFPGER and SSGDQGPAG, alongside SSGPFGE, exhibited predominantly umami, sweet, and mildly sour profiles, constituting a positive taste spectrum. Conversely, LPGADDDKF and GLPGADDKF exhibited pronounced negative taste characteristics including bitterness and astringency, with significantly diminished positive flavour contributions. Further quantitative analysis of umami intensity in Fig. 7C and Fig. 7D demonstrated high consistency between electronic tongue and sensory evaluations regarding umami trends, with all peptides reaching peak umami intensity at a mass concentration of 20 mg/mL. GDRGFPGER exhibited the highest umami intensity at 20 mg/mL. When blended with monosodium glutamate (MSG) solutions, SSGDQGPAG and SSGPFGE significantly enhanced umami intensity (*P* < 0.05). LPGADDDKF and GLPGADDKF not only exhibited weak umami profiles themselves, but their pronounced bitter taste significantly inhibited the inherent umami of MSG, highlighting the negative regulatory effect of adverse tastes on overall flavour quality. Among positive taste dominant peptides, umami intensity ranked as follows: GDRGFPGER > SSGDQGPAG > SSGPFGE > MSG > LPGADDDKF > GLPGADDKF. SSGDQGPAG and SSGPFGE, as umami enhancing peptides, possess the capacity to supplement or amplify the inherent flavour of foods, potentially stimulating increased consumer appetite (Xiang et al., 2025). However, LPGADDDKF and GLPGADDKF exhibit pronounced bitterness and are unsuitable as umami enhancers. The umami intensity of GDRGFPGER is significantly higher than that of other umami peptides, consistent with results from dynamic molecular dynamics simulations and relevant published studies (Chen et al., 2025; Lao et al., 2025).

## 4 Conclusion

In summary, this study utilised hydrolysed cod skin as raw material. Through ultrafiltration separation, LC–MS/MS mass spectrometry identification, virtual screening combined with static molecular docking and dynamic molecular dynamics simulations, five non toxic, water soluble, and bioactive potential umami peptides: GDRGFPGER, SSGDQGPAG, SSGPFGE, LPGADDKF, and GLPGADDKF. Five candidates formed stable static interactions with T1R1/T1R3 subunits and the umami heterodimeric receptor. Dynamic docking stability validation revealed poor conformational stability in molecular dynamics simulations for SSGPFGE, LPGADDKF, and GLPGADDKF. Combining electronic tongue taste profiles with sensory umami intensity assessments ultimately identified GDRGFPGER as possessing the highest umami intensity, confirming it as the optimal umami peptide. These findings strengthen the theoretical basis for identifying potential umami peptides in hydrolysed cod skin products, broaden our understanding of the molecular mechanisms governing umami flavour presentation, and lay the groundwork for their application in sensory enhancement and health promotion within food formulations.

## CRediT authorship contribution statement

**Shengli Shi:** Writing – review & editing, Resources. **Mengkai Li:** Writing – original draft, Methodology, Conceptualization. **Yiqing Wang:** Software, Methodology. **Dan Li:** Software. **Tiantian Wang:** Software. **Aiguo Luo:** Project administration, Funding acquisition. **Hongmei Zhao:** Investigation. **Xiaoli Ma:** Software. **Yanjun Yang:** Resources, Funding acquisition.

## Institutional review board statement

The food sensory evaluation study involved in this research has been reviewed and formally approved by the Human Research Ethics Committee of Shanxi University (approval no. SXULL2026132). All experimental procedures were conducted in accordance with the ethical principles set forth in the Declaration of Helsinki. Written informed consent was obtained from each panelist prior to their participation in the sensory evaluation.

## Declaration of competing interest

The authors declare that they have no known competing financial interests or personal relationships that could have appeared to influence the work reported in this paper.

## Data Availability

No data was used for the research described in the article.
