# Supplementary material for: Identification and characterization of umami peptides from enzymatic hydrolysates of cod skin based on sensory evaluation and molecular simulations
Source: Food Chem X. 2026 Jul 23;38:104245. doi: 10.1016/j.fochx.2026.104245 (PMC13450613; doi:10.1016/j.fochx.2026.104245)
Supplement: Supplementary file 1 [file mmc1.docx]

**Supplementary materials**

**Identification and characterization of umami peptides from enzymatic hydrolysates of cod skin based on sensory evaluation and molecular simulations**

Shengli Shi^a^, Mengkai Li^b^, Yiqing Wang^b^, Dan Li^b^, Tiantian Wang^b^, Aiguo Luo^b^, Hongmei Zhao^a^, Xiaoli Ma^a^, Yanjun Yang^a[1]*^

*a: Department of Biological Science and Technology, Jinzhong University, Jinzhong 030619, China;*

*b: Shanxi Composite Condiment Technology Innovation Center, Jinzhong 030619, China;*

**Table S1** Classification criteria for taste intensity based on electronic tongue response values

| Flavour | Grading | Score Range | Description |
| --- | --- | --- | --- |
| Sour/Sweet/Salty/Savoury/Bitter | Tasteless | 0~100 | No |
|  | Faint flavour | 101~300 | Weak |
|  | Moderate flavour | 301~500 | Moderate |
|  | Strong flavour | 501~700 | Strong |
|  | Very strong flavour | 701~900 | Saturated |
| Astringency/Astringent aftertaste/Bitter aftertaste | No aftertaste | 0~100 | No |
|  | Faint aftertaste | 101~300 | Weak |
|  | Moderate aftertaste | 301~500 | Moderate |
|  | Strong aftertaste | 501~700 | Strong |
|  | Very strong aftertaste | 701~900 | Saturated |

**Table S2** Classification criteria for taste intensity based on sensory evaluation

| Level | Score | Description |
| --- | --- | --- |
| Taste intensity level | 1 | No discernible flavour, no taste signals in the mouth |
| No taste | 2~3 | Barely perceptible faint flavour, weak and unstable signals |
| Faint taste | 4~5 | Clear perception of flavour, stable signals, moderate intensity, high distinguish ability |
| Moderate taste | 6~7 | Pronounced flavour intensity, strong and persistent signals, clear and prominent oral taste feedback |
| Strong taste | 8~10 | Extremely intense flavour, saturated and stimulating signals, intense and prolonged oral taste feedback |

**Table S3** Umami response and sensory umami score of different samples

| Samples | Electronic tongue umami response | Sensory umami score |
| --- | --- | --- |
| F1 | 645.33 ± 6.03^a^ | 7.54 ± 0.05^a^ |
| F2 | 512 ± 4.58^b^ | 5.88 ± 0.08^b^ |
| F3 | 313.67 ± 4.04^c^ | 3.05 ± 0.37^c^ |
| F4 | 102 ± 3.61^d^ | 0.87 ± 0.07^d^ |

Results are presented as mean ± standard deviation. Different superscript letters within the same column indicate significant differences (p < 0.05). Electronic tongue umami responses are expressed in arbitrary units (a.u.); sensory umami scores were evaluated on a 10-point scale and standardized

Table S4 Parameters of T1R1/T1R3 active binding pocket

| pockets | Volume / Å^3^ | Surface area / Å^2^ | Depth / Å | Hydrogen bond donor | Hydrogen bond acceptor | Drug Binding Capacity Score | Score |
| --- | --- | --- | --- | --- | --- | --- | --- |
| T1R1 | 1748.91 | 1894.35 | 27.6 | 46 | 117 | 0.81 | 0.60 |
| T1R3 | 1608.32 | 1707.08 | 29.79 | 32 | 92 | 0.80 | 0.61 |

Table S5 Key amino acid residues for molecular docking between potential umami peptides and T1R1/T1R3 receptors

| Binding site | GDRGFPGER | SSGDQGPAG | SSGPFGE | LPGADDKF | GLPGADDKF |
| --- | --- | --- | --- | --- | --- |
| T1R1 |  |  |  |  |  |
| Arg56 | + | ++ |  | ++ | ++ |
| Ser109 |  | + | + | + | ++ |
| Ala249 | ++ | + | + |  |  |
| Ser148 | + |  | + |  | + |
| Asp108 |  | + |  |  | + |
| Asp218 | + | + | + | + | + |
| Ser107 | ++ | ++ | + |  | ++ |
| Ser276 |  |  | + |  | ++ |
| Gln278 | + |  |  |  | ++ |
| Ser248 | ++ | ++ | + | + | + |
| Phe247 | + | + | + | ++ | + |
| Arg151 | +++ | +++ | ++ | ++ | +++ |
| Asn150 |  | + | + | + |  |
| T1R3 |  |  |  |  |  |
| Gly84 | ++ |  |  | + |  |
| Ser306 | ++++ |  | ++ | + |  |
| Leu468 | ++ |  | ++ | ++ |  |
| Asn386 | ++ |  | ++ | + |  |
| Asp470 |  |  |  | + |  |
| Gln330 | ++ |  | ++ | ++ |  |
| His387 | + |  | + |  |  |
| Lys457 | + |  |  | + |  |
| Asp455 | + |  |  |  |  |
| Gly328 | ++ |  | + |  |  |
| Phe391 | + |  | ++ |  |  |
| Thr390 |  |  | + |  |  |
| Leu331 | + |  | + |  |  |

Table S6 Key amino acid residues in molecular docking of umami peptides with T1R1/T1R3 heterodimer

| Binding site | GDRGFPGER | SSGDQGPAG | SSGPFGE | LPGADDKF | GLPGADDKF |
| --- | --- | --- | --- | --- | --- |
| Chain A | | | | | |
| Ser48 |  | ++ |  |  | + |
| Phe247 | + | + | + | ++ | +++ |
| Ser248 | ++ | ++ | + |  | +++++ |
| Ala249 | + | ++ | + |  | + |
| Asp218 | + |  | + |  | + |
| Asn150 | ++ | + | + | + | + |
| Ser217 | + | ++ | ++ |  | + |
| Ser107 |  | ++ | ++ |  |  |
| Gln52 |  |  |  | + | + |
| Arg151 | +++ | ++ | ++ | + | ++ |
| Chain B | | | | | |
| Leu173 | + |  | + | + | +++ |
| Thr179 | ++ | + | + | ++ |  |
| Lys155 | + | + | + | ++ | + |
| Ala176 |  |  | + | + |  |
| Glu217 |  |  | + |  |  |
| Val152 |  |  |  | + |  |
| Phe180 | ++ |  |  |  |  |

1. * Corresponding author: E–mail address: yyj1210@sina.com
